# Optimized inferences of finite population mean using robust parameters in systematic sampling

**DOI:** 10.1371/journal.pone.0278619

**Published:** 2023-01-23

**Authors:** Nazia Shaheen, Muhammad Nouman Qureshi, Osama Abdulaziz Alamri, Muhammad Hanif

**Affiliations:** 1 Department of Statistics, National College of Business Administration and Economics, Lahore, Pakistan; 2 School of Statistics, University of Minnesota, Minnesota, Minneapolis, United States of America; 3 Department of Statistics, University of Tabuk, Tabuk, Saudi Arabia; University of Bradford, UNITED KINGDOM

## Abstract

In this article, we have proposed a generalized estimator for mean estimation by combining the ratio and regression methods of estimation in the presence of auxiliary information using systematic sampling. We incorporated some robust parameters of the auxiliary variable to obtain precise estimates of the proposed estimator. The mathematical expressions for bias and mean square error of proposed the estimator are derived under large sample approximation. Many other generalized ratio and product-type estimators are obtained from the proposed estimator using different choices of scalar constants. Some special cases are also discussed in which the proposed generalized estimator reduces to the usual mean, classical ratio, product, and regression type estimators. Mathematical conditions are obtained for which the proposed estimator will perform more precisely than the challenging estimators mentioned in this article. The efficiency of the proposed estimator is evaluated using four populations. Results showed that the proposed estimator is efficient and useful for survey sampling in comparison to the other existing estimators.

## 1. Introduction

In survey sampling, it is well known that no single estimation technique can always provide the best results for populations of different characteristics under different situations. In many real-life situations, the researchers may incline to use such sampling techniques which can provide better estimation results in limited time, cost, and effort. Compared to the other sampling designs, systematic sampling is often considered to be a better choice, as it is easy to apply and can provide increased precision of the estimators. In systematic sampling, the units are selected as per some criterion after selecting the first unit at random. Madow and Madow [1] first studied the theory of systematic sampling and considered it the most frequently used probability sampling design for population parameter estimation due to its easiness. Cochran [2] reviewed the applications of systematic sampling and concluded that “apart from being easy to implement, systematic sampling provides more efficient estimators as compared to the simple random sampling or stratified sampling for certain types of populations”. Finney [3] and Zinger [4] discussed the application of systematic sampling for natural populations like forests. Cochran [5] cited many applications for systematic sampling in forestry, agriculture, and land surveys, and suggested that it can provide implicit stratification to produce better estimates for the situation when the sampling frame is in some specific order.

In survey sampling, the researchers collect information on the variable(s) that are correlated (either positively or negatively) with the main variable of interest. The use of such variables(s) along with the main study variable is very common to improve the efficiency of the estimator(s) for the population parameter(s) such as the mean, total, variance, proportion, etc. Usually, the ratio-type estimators are considered if the correlation is positive and the product-type estimators may be used when the correlation is negative between the study and the auxiliary variables. Moreover, many authors used some conventional and non-conventional parameters of the auxiliary variables to increase the precision of the estimators. Several authors have used systematic sampling for the situation when the auxiliary information was available with the concerned variable. Swain [6] suggested a ratio estimator whereas Shukla [7] proposed the product estimator using systematic sampling. Singh [8] suggested a ratio-cum-product type estimator in systematic sampling design. Srivastava and Jhajj [9] proposed a class of estimators for population mean using multi-auxiliary variables whereas Kushwaha and Singh [10] recommended a family of almost unbiased ratio and product estimators in systematic sampling. Banarasi *et al*. [11] proposed a family of ratio, product, and difference-type estimators using systematic sampling. Singh and Singh [12] proposed unbiased ratio and product type estimators in systematic sampling. Recently, some estimators were proposed using the exponent of the auxiliary variable by Singh *et al*. [13], Singh and Jatwa [14], Singh and Solanki [15], Tailor *et al*. [16], Khan and Singh [17], Khan [18] and Qureshi *et al*. [19].

In this article, we have proposed a generalized mean estimator using the auxiliary information with the expectation that the proposed estimator will perform better than the competing estimators. We have used some robust parameters associated with the auxiliary variable such as coefficient of kurtosis (*β*_*2x*_), upper quartile (*Q*_*3x*_), mid-range (*M*_*x*_), Hodges-Lehmann (*H*_*x*_), tri-mean (*T*_*x*_), Gini’s mean difference (*G*_*x*_), Downton’s method (*D*_*x*_), probability-weighted moments (*P*_*x*_) and deciles mean (*DM*_*x*_). The generalized estimator also produces a family of sub-generalized estimators and sub-families of ratio and product-type estimators. In Section 2, we have discussed sampling methodology, notations, and some associated estimators. In Section 3, we derived the expressions for bias and *MSE* of the proposed estimator. Many special cases have been discussed in which the proposed estimator reduces to ratio and product-type estimators. Mathematical comparisons of the proposed estimator with the existing estimators are given in the same Section. An extensive numerical study is conducted using two real and two artificial populations in Section 4. A brief discussion of the paper is given in Section 5.

## 2. The methodology of systematic sampling and classical estimators

Consider a population *P* = (*P*_*1*_, *P*_*2*_, *…*, *P*_*j*_,*…*, *P*_*N*_) composed of *N* distinct elements in some specific order that labeled from 1 to *N* (*1*,*2*, *…*,*j*, *…*, *N*), where we mentioned the *j*^*th*^ element on *P* denoted by *P*_*j*_ for the simplicity. Further suppose that *N* is a product of two non-negative integers *n* and *k*, such that *N* = *nk*. Let *Y* is the main variable of interest and *X* be an auxiliary variable for which the values of both the study and the auxiliary variables can be defined by their label as *y* = (*y*_*1*_, *y*_*2*_,*…*,*y*_*j*_,*…*, *y*_*N*_) and *x* = (*x*_*1*_, *x*_*2*_,*…*,*x*_*j*_,*…*, *x*_*N*_) respectively. Let *y*_*ij*_ and *x*_*ij*_ be the values of the main study variable and the auxiliary variable on *j*^*th*^ (*j* = *1*, *2*, *3*, *…*, *k*) unit in the *i*^*th*^ (*i* = *1*, *2*, *3*, *…*, *n*) systematic sample. To select a sample of size *n*, we draw a random number between 1 to *k* (suppose it is *j*) and then every *k*^*th*^ unit is selected such that *j*, *j+k*, *j+2k*, *…*, *j+*(n-1) *k* successive digits. Consequently, we have total *k* possible samples for each size *n*.

The means and variances for the study and the auxiliary variables for systematic samples may be obtained as

$${\bar{y}}_{sys}=\sum_{j=1}^{n}y_{ij}/n$$

and

$${\bar{x}}_{sys}=\sum_{j=1}^{n}x_{ij}/n\cdot$$

$$s_{y}^{2}=\sum_{j=1}^{n}{\left(y_{ij}-\bar{y}\right)}^{2}/(n-1)$$

and

$$s_{x}^{2}=\sum_{j=1}^{n}{\left(x_{ij}-\bar{x}\right)}^{2}/(n-1).$$

The mean estimator (without having the auxiliary information) along with the variance equation is given by

$${\hat{\mu }}_{0,sys}={\bar{y}}_{sys}.$$

$$var\left({\hat{\mu }}_{0,sys}\right)=\theta \rho_{y}^{*}\mu_{y}^{2}C_{y}^{2}.$$

where

$$\rho_{y}^{*}=\left(1+\left(n-1\right)\rho_{y}\right),\mu_{y}=N^{-1}\sum_{i=1}^{N}y_{ij},\sigma_{y}^{2}={\left(N-1\right)}^{-1}\sum_{i=1}^{k}{\sum_{j=1}^{n}\left(y_{ij}-\mu_{y}\right)}^{2},$$

$$C_{y}=\sigma_{y}/\mu_{y},\rho_{y}=\left[E\left(y_{ij}-\mu_{y}\right)\left(y_{ij^{\prime }}-\mu_{y}\right)\right]/E{\left(y_{ij}-\mu_{y}\right)}^{2}$$

and

θ=(N−1)/Nn.

The ratio and product estimators proposed by Swain (1964) and Shukla (1971) under systematic sampling design are defined as

$${\hat{\mu }}_{R,sys}=\frac{{\bar{y}}_{sys}}{{\bar{x}}_{sys}}\mu_{x}.$$

$${\hat{\mu }}_{P,sys}=\frac{{\bar{y}}_{sys}}{\mu_{x}}{\bar{x}}_{sys}.$$

The expressions for *MSE* for the estimators *µ*_*r*, *sys*_ and *µ*_*p*,*sys*_ are given by

$$MSE\left({\hat{\mu }}_{r,sys}\right)\approx \theta {\bar{Y}}^{2}\left(\rho_{y}^{*}C_{y}^{2}+\rho_{x}^{*}C_{x}^{2}\left(1-2H_{yx}\sqrt{\rho_{y/x}^{*}}\right)\right).$$

$$MSE\left({\hat{\mu }}_{p,sys}\right)\approx \theta {\bar{Y}}^{2}\left(\rho_{y}^{*}C_{y}^{2}+\rho_{x}^{*}C_{x}^{2}\left(1+2H_{yx}\sqrt{\rho_{y/x}^{*}}\right)\right),$$

where

$$\rho_{x}^{*}=\left(1+\left(n-1\right)\rho_{x}\right),\rho_{x}=\left[E\left(x_{ij}-\mu_{x}\right)\left(x_{ij\prime }-\mu_{x}\right)\right]/E{\left(x_{ij}-\mu_{x}\right)}^{2},C_{x}=\sigma_{x}^{}/\mu_{x}^{}$$

where

$$\mu_{x}=N^{-1}\sum_{i=1}^{N}x_{ij}$$

$$\sigma_{x}^{2}={\left(N-1\right)}^{-1}\sum_{i=1}^{k}{\sum_{j=1}^{n}\left(x_{ij}-\mu_{x}\right)}^{2},$$

$$\rho_{y/x}^{*}=\rho_{y}^{*}/\rho_{x}^{*},\rho_{x/y}^{*}=\rho_{x}^{*}/\rho_{y}^{*}$$

and

$$H_{yx}=\rho_{yx}C_{y}/C_{x}.$$

Here *ρ*_*y*_ and *ρ*_*x*_ are the intra-class correlations whereas *C*_*y*_ and *C*_*x*_ are the coefficients of correlation of their subscripts and *ρ*_*yx*_ is the correlation coefficient between the subscript.

## 3. Proposed generalized estimator

In this section, we have proposed a generalized estimator using an auxiliary variable for the estimation of population mean by combining modified ratio and regression type estimators under systematic sampling as

$${\hat{\mu }}_{pi,sys}^{\left(G\right)}=\left({\bar{y}}_{sys}+\frac{b_{sys}\left(\mu_{x}-{\bar{x}}_{sys}\right)}{\mu_{x}}\right){\left(\frac{\mu_{z}}{\lambda {\bar{z}}_{sys}+(1-\lambda )\mu_{z}}\right)}^{v},$$ (1)

With

$${\bar{z}}_{sys}=\alpha {\bar{x}}_{sys}+\gamma$$

and

$$\mu_{z}=\alpha \mu_{x}+\gamma ,$$

where *b*_*sys*_ is the regression coefficient between the sample observations of the study variable and the auxiliary variable and *v* is a suitable constant to produce ratio type and product type estimators by assuming values 1 and -1 respectively. Here λ is an optimized constant used for the value of minimum *MSE*. Further, we have *α* (*α≠* 0) and *γ*, which may assume real numbers or different robust parameters (define in Appendix *E*) of the auxiliary variable *X*.

Some notations are necessary to derive the bias and the *MSE*. Let

$${\bar{y}}_{sys}=\mu_{y}\left(1+\xi_{y}\right),{\bar{x}}_{sys}=\mu_{x}\left(1+\xi_{x}\right),s_{yx}=\sigma_{yx}\left(1+\xi_{s_{yx}}\right)$$

and

$$s_{x}^{2}=\sigma_{x}^{2}\left(1+\xi_{s_{x}^{2}}\right),$$

$$E\left(\xi_{y}\right)=E\left(\xi_{x}\right)=E\left(\xi_{\beta }\right)=0,E\left(\xi_{y}^{2}\right)=\theta \rho_{y}^{*}C_{y}^{2},$$

$$E\left(\xi_{x}^{2}\right)=\theta \rho_{y}^{*}C_{x}^{2}$$

and

$$E\left(\xi_{y}\xi_{x}\right)=\theta C_{x}^{2}\sqrt{\rho_{y/x}^{*}}H_{yx}.$$

Using the above notations, the proposed generalized estimator in (1) may be written as

$${\hat{\mu }}_{pi,sys}^{\left(G\right)}=\left(\mu_{y}\left(1+\xi_{y}\right)+\frac{\sigma_{yx}\left(1+\xi_{s_{yx}}\right)}{\sigma_{x}^{2}\left(1+\xi_{s_{x}^{2}}\right)}\frac{\left(\mu_{x}-\mu_{x}\left(1+\xi_{x}\right)\right)}{\mu_{x}}\right){\left(\frac{\alpha \mu_{x}+\gamma }{\lambda \left(\alpha \mu_{x}\left(1+\xi_{x}\right)+\gamma \right)+(1-\lambda )\left(\alpha \mu_{x}+\gamma \right)}\right)}^{v}.$$ (2)

Simplifying and expanding the above equation using the Taylor series up to the first order, we have

$${\hat{\mu }}_{pi,sys}^{\left(G\right)}\approx \left(\mu_{y}+\mu_{y}\xi_{y}-\beta_{sys}\left(\xi_{x}+\xi_{x}\xi_{s_{yx}}-\xi_{x}\xi_{s_{x}^{2}}\right)\right)\left(1-\lambda v\psi_{j}\xi_{x}\right),$$

where

$$\beta_{sys}=\sigma_{yx}/\sigma_{x}^{2}$$

and

$$\psi_{j}=\alpha \mu_{x}/\left(\alpha \mu_{x}+\gamma \right)$$

After simplification, we applied expectation to the above equation, and we get

$$E\left({\hat{\mu }}_{pi,sys}^{\left(G\right)}-\mu_{y}\right)\approx \left(\theta \lambda v\psi_{j}\beta_{sys}\rho_{x}^{*}C_{x}^{2}-\theta \mu_{y}\lambda v\psi_{j}\sqrt{\rho_{y}^{*}\rho_{x}^{*}}\rho_{yx}C_{y}C_{x}\right)-\beta_{sys}\left(\frac{\theta \mu_{12}}{\mu_{x}\mu_{11}}-\frac{\theta \mu_{03}}{\mu_{x}\mu_{02}}\right).$$

The mathematical expression of bias of the proposed estimator is

$$Bias\left({\hat{\mu }}_{pi,sys}^{\left(G\right)}\right)\approx \theta \left[\lambda v\psi_{j}\rho_{x}^{*}C_{x}^{2}\left(\beta_{sys}-\mu_{y}\sqrt{\rho_{y/x}^{*}}H_{yx}\right)-\mu_{x}^{-1}\beta_{sys}\left(\mu_{11}^{-1}\mu_{12}-\mu_{02}^{-1}\mu_{03}\right)\right],$$

where

$$\mu_{rs}={\left(N-1\right)}^{-1}\sum_{i=1}^{N}{\left(Y_{i}-\mu_{y}\right)}^{r}{\left(X_{i}-\mu_{x}\right)}^{s}$$

r,s=0,1,2,3.

We re-write (2) by using Taylor expansion up to the first-order

$${\hat{\mu }}_{pi,sys}^{\left(G\right)}-\mu_{y}\approx \mu_{y}\xi_{y}-\left(\beta_{sys}+\mu_{y}\lambda v\psi_{j}\right)\xi_{x}.$$

On squaring and applying the expectation of the above equation, we get

$$E{\left({\hat{\mu }}_{pi,sys}^{\left(G\right)}-\mu_{y}\right)}^{2}\approx E\left(\mu_{y}^{2}\xi_{y}^{2}+{\left(\beta_{sys}+\mu_{y}\lambda v\psi_{j}\right)}^{2}\xi_{x}^{2}-2\mu_{y}\left(\beta_{sys}+\mu_{y}\lambda v\psi_{j}\right)\xi_{y}\xi_{x}\right).$$

The first-order *MSE* of the proposed estimator is

$$MSE\left({\hat{\mu }}_{pi,sys}^{\left(G\right)}\right)\approx \theta \left(\mu_{y}^{2}\rho_{y}^{*}C_{y}^{2}+\left(\beta_{sys}+\mu_{y}\lambda v\psi_{j}\right)\rho_{x}^{*}C_{x}^{2}\left\{\left(\beta_{sys}+\mu_{y}\lambda v\psi_{j}\right)-2\mu_{y}\sqrt{\rho_{y/x}^{*}}H_{yx}\right\}\right).$$ (3)

### 3.1 Optimum choice for scalar “*λ*”

For the minimum *MSE* of the proposed estimator, we differentiate (3) for “*λ*” and equate to zero to get the optimum value of scalar *λ* as

$$\lambda_{opt}=\frac{1}{v\psi_{j}}\left[\sqrt{\rho_{y/x}^{*}}H_{yx}-\mu_{j}^{-1}\beta_{sys}\right].$$

The minimum *MSE* of the proposed estimator is given by

$$MSE_{min}\left({\hat{\mu }}_{yi,sys}^{\left(G\right)}\right)\approx \theta \rho_{y}^{*}S_{y}^{2}\left(1-\rho_{yx}^{2}\right).$$

The $MSE_{min}\left({\hat{\mu }}_{pi,sys}^{\left(G\right)}\right)$ can be obtained by replacing $S_{y}^{2}$, $\rho_{y}^{*}$, *ρ*_*yx*_ and *λ*_*opt*_ their estimates $S_{y}^{2}$, $r_{y}^{*}$, *r*_*yx*_ and ${\hat{\lambda }}_{opt}$ respectively.

Note that the proposed estimator may reduce to some classical estimators using different values of *b*_*sys*_, *v*, *α*, *γ*, and *λ* as shown in Table 1.

**Table 1:** Classical estimators as the family of the proposed estimator.

*Type*	*Estimators*	*b*_*sys*_	*v*	*α*	*γ*	*λ*
*Mean Estimator*	${\hat{\mu }}_{y,sys}={\bar{y}}_{sys}$	0	0	-	-	-
*Ratio Estimator*	${\hat{\mu }}_{R,sys}={\bar{y}}_{sys}\left(\mu_{x}/{\bar{x}}_{sys}\right)$	0	1	1	0	1
*Regression type Estimator*	${\hat{\mu }}_{r,sys}={\bar{y}}_{sys}+b_{sys}\left(\mu_{x}-{\bar{x}}_{sys}\right)/\mu_{x}$	*b*_*sys*_	0	-	-	-
*Difference type Estimator*	* ${\hat{\mu }}_{d,sys}={\bar{y}}_{sys}+d\left(\mu_{x}-{\bar{x}}_{sys}\right)/\mu_{x}$ *	*d*	0	-	-	-
*Product Estimator*	${\hat{\mu }}_{P,sys}={\bar{y}}_{sys}\left({\bar{x}}_{sys}/\mu_{x}\right)$	0	-1	1	0	1
*Regression-ratio Estimator*	${\hat{\mu }}_{rr,sys}=\left[{\bar{y}}_{sys}+b_{sys}\left(\mu_{x}-{\bar{x}}_{sys}\right)/\mu_{x}\right]\left(\mu_{x}/{\bar{x}}_{sys}\right)$	*b*_*sys*_	1	1	0	1
*Regression-product Estimator*	* ${\hat{\mu }}_{rp,sys}=\left[{\bar{y}}_{sys}+b_{sys}\left(\mu_{x}-{\bar{x}}_{sys}\right)/\mu_{x}\right]\left({\bar{x}}_{sys}/\mu_{x}\right)$ *	*b*_*sys*_	-1	1	0	1

### 3.2 Algebraic comparisons

In this sub-section, we have made some algebraic comparisons to get the optimum conditions in which the proposed estimator performs superior to the competing estimators.

The proposed estimator performs better than the basic mean estimator if

$$MSE_{min}\left({\hat{\mu }}_{pi,sys}^{\left(G\right)}\right)<var\left({\hat{\mu }}_{0,sys}\right).$$

or

$$\rho_{yx}^{2}>0.$$

The proposed estimator performs better than the usual ratio estimator if

$$MSE_{{}_{min}}\left({\hat{\mu }}_{pi,sys}^{\left(G\right)}\right)<MSE\left({\hat{\mu }}_{R,sys}\right).$$

or

$$\rho_{yx}^{2}>\rho_{x/y}^{*}\left(2H_{yx}\sqrt{\rho_{y/x}^{*}}-\frac{C_{x}^{2}}{C_{y}^{2}}\right).$$

The proposed estimator performs better than the product-type estimator if

$$MSE_{min}\left({\hat{\mu }}_{pi,sys}^{\left(G\right)}\right)<MSE\left({\hat{\mu }}_{P,sys}\right).$$

or

$$\rho_{yx}^{2}>-\rho_{x/y}^{*}\left(\frac{C_{x}^{2}}{C_{y}^{2}}-2H_{yx}\sqrt{\rho_{y/x}^{*}}\right).$$

## 4. Simulation study

For the assessment of the proposed estimator and its sub-cases, a simulation study is carried out for each the population separately. The statistics of all the populations are presented in Appendix *B*. The mathematical formulae to compute the absolute biases (*ABs*) and percentage relative efficiencies (*PREs*) for all the estimators are defined as

$$AB\left(Est\right)=\frac{1}{50,000}\left|\sum_{i=1}^{50,000}\left(Est-\mu_{y}\right)\right|,$$

and

$$PRE\left(Est\right)=\frac{var\left({\bar{y}}_{sys}\right)}{M\hat{S}E\left(Est\right)}\text{x}100,$$

where

$$M\hat{S}E\left(Est\right)=\frac{1}{50,000}\sum_{i=1}^{50,000}{\left(Est-\mu_{y}\right)}^{2}.$$

$$Est={\hat{\mu }}_{0,sys},{\hat{\mu }}_{R,sys},{\hat{\mu }}_{P,sys},{\hat{\mu }}_{p1,sys}^{\left(*\right)},{\hat{\mu }}_{p2,sys}^{\left(*\right)},\ldots ,{\hat{\mu }}_{p11,sys}^{\left(*\right)}$$

and

$${\hat{\mu }}_{pi,sys}^{\left(G\right)}.$$

*=AandF.

### Sources of population

Four populations have been taken to obtain the results of *ABs* and *PREs* of all the estimators using 50,000 simulations independently. The sources of the population are

**Source-1:**
Cochran [5]
*Y*= Food Cost; *X*= No. of persons in a family
*N*=33 and *n* = 03.
**Source-1I:**
Murthy [20]
*Y* = Timber Volume; *X* = Strip-wise Length (cents)
*N* = 176 and *n* = 24.
**Sources-III:**
A bivariate population for (*Y*, *X*) is generated using an *R*-package of uniform distribution with the parameters 2 and 50.
$$\begin{array}{l} X=2\text{runif}\left(1000,2,50\right).\\ Y=4X+2\text{runif}\left(1000,2,50\right). \end{array}$$
N=1000andn=200.
**Source-IV:**
  A bivariate normal population of size *N* = 1000 (*n* = 200) is generated using an *R*-package with a mean vector and variance-covariance matrix:
$$\mu ={\left[\begin{array}{cc} 5 & 5 \end{array}\right]}^{/}$$
  and
$$\sum =\left[\begin{array}{cc} 5 & -3\\ -3 & 2 \end{array}\right]$$

The following steps have been coded in *R*-language to get the results of *ABs* and the *PREs*.


Step-1Fromthedescribedpopulations,wehavecomputedthepopulationmeanoftheauxiliaryvariablex.Step-2Weconsiderdifferentsamplesizesforeachpopulation,togeneratethesamplesthatfollowthesystematicpatterni.e.,j,j+k,j+2k,j+3k,…,j+(n−1)k.Step-3Foreachsamplesize,thevaluesofABsandPREsarecomputedforalltheestimatorsconsideredinthispaper.Step-4TheprocessinStep-2andStep-3isrepeated50,000timesandtheoutcomesarereportedinTable2

From the numerical illustrations presented in Table 2, it is notice that the generalized estimator is more efficient than the competing estimators based on the results of *ABs* and *PREs* for all populations. Many special cases of ratio-type ($t_{pi,sys}^{\left(A\right)}$ and $t_{pi,sys}^{\left(F\right)}$) estimators are computed for populations I-III and product-type ($t_{pi,sys}^{\left(B\right)}$ and $t_{pi,sys}^{\left(H\right)}$) estimators for population IV (Appendix *C*). We have used *v* = 1 for populations I-III and *v* = -1 for population IV and observed that the ratio-type estimators performed well for the populations having positive correlation whereas the product-type estimators performed better for the population having negatively correlated auxiliary data with the study variable. Overall results revealed that the efficiency of the proposed estimator is every high than existing estimators for all populations.

**Table 2:** The amount of *ABs* and *PREs* of different estimators.

Estimators	*Absolute Biases*				*Percent Relative Efficiencies*			
	*Pop-I* *ρ*_*yx*_ *= 0*.*43*	*Pop-II* *ρ*_*yx*_ *= 0*.*87*	*Pop-III* *ρ*_*yx*_ *= 0*.*98*	*Pop-IV* *ρ*_*yx*_ *= -0*.*95*	*Pop-I* *ρ*_*yx*_ *= 0*.*43*	*Pop-II* *ρ*_*yx*_ *= 0*.*87*	*Pop-III* *ρ*_*yx*_ *= 0*.*98*	*Pop-IV* *ρ*_*yx*_ *= -0*.*95*
${\hat{\mu }}_{0,sys}^{}$	0.0002	0.0003	0.0015	0.0019	100.000	100.000	100.000	100.000
${\hat{\mu }}_{R,sys}^{}$	0.177	0.019	0.005	0.070	103.562	309.668	1938.138	39.191
${\hat{\mu }}_{P,sys}^{}$	0.300	0.067	0.041	0.019	25.466	45.487	21.564	604.125
${\hat{\mu }}_{pi,sys}^{\left(G\right)}$	0.109	0.011	0.002	0.016	177.126	414.331	4654.115	1031.549

## 5. Conclusion

Systematic sampling is often considered to be very useful for populations under different disciplines like agriculture, environmental, ecological, forestry, and marine science. It is also applied outside the afford-mention fields, as it is easy to understand and simple to execute. In the present study, our primary concern is to suggest a generalized estimator for the estimation of population mean using systematic sampling. The proposed estimator can produce several other estimators as special cases using different choices of defining parameters. We further suggested several efficient estimators as special cases of the proposed estimator using known parameters associated with the auxiliary variable. The optimum conditions are also derived under which the proposed estimator provide more precise estimates as compared to the existing estimators. We have analyzed the numerical comparison using two real and two artificial populations. The proposed estimator along with its sub-estimators has shown great efficiency than the sample mean, classical ratio, and product estimators for all populations. The proposed estimator performs better than the ratio and product estimator for *v* = 1, and -1. On the behalf of theoretical comparisons and numerical findings, we suggest that the proposed estimator is very efficient and useful for mean estimation using systematic sampling.

Only single auxiliary variable is considered in this research to propose the generalized estimator for mean estimation. In future, multi auxiliary variables would be incorporated to propose more generalized estimator in the presence of measurement error and nonresponse using systematic sampling.

## Supporting information

S1 Appendix This file contains multiple tables. (DOCX)
