# Supplementary material for: Optimized inferences of finite population mean using robust parameters in systematic sampling
Source: PLoS One. 2023 Jan 23;18(1):e0278619. doi: 10.1371/journal.pone.0278619 (PMC9870168; doi:10.1371/journal.pone.0278619)
Supplement: S1 Appendix — (DOCX) [file pone.0278619.s001.docx]

**Appendices**

**Appendix A**

**Table A1: Some Generalized Estimators using different Choices of Constants**

| ***Ratio type Estimators*** | ***Product Type Estimator*** |  |  |
| --- | --- | --- | --- |
| *v = 1* | *v = -1* | *$b_{yx}$* | $\lambda$ |
| *$\hat{\mu}_{pi,sys}^{\left(A\right)}=\bar{y}_{sys}\left(\frac{\mu_{z}}{\bar{z}_{sys}}\right)$* | *$\hat{\mu}_{pi,sys}^{\left(B\right)}=\bar{y}_{sys}\left(\frac{\bar{z}_{sys}}{\mu_{z}}\right)$* | *0* | *1* |
| *$\hat{\mu}_{pi,sys}^{\left(D\right)}=\left[\bar{y}_{sys}+\frac{\left(\mu_{x}-\bar{x}_{sys}\right)}{\mu_{x}}\right]\left(\frac{\mu_{z}}{\bar{z}_{sys}}\right)$* | *$\hat{\mu}_{pi,sys}^{\left(E\right)}=\left[\bar{y}_{sys}+\frac{\left(\mu_{x}-\bar{x}_{sys}\right)}{\mu_{x}}\right]\left(\frac{\bar{z}_{sys}}{\mu_{z}}\right)$* | *1* | *1* |
| *$\hat{\mu}_{pi,sys}^{\left(F\right)}=\left[\bar{y}_{sys}+\frac{b_{yx}\left(\mu_{x}-\bar{x}_{sys}\right)}{\mu_{x}}\right]\left(\frac{\mu_{z}}{\bar{z}_{sys}}\right)$* | *$\hat{\mu}_{pi,sys}^{\left(H\right)}=\left[\bar{y}_{sys}+\frac{b_{yx}\left(\mu_{x}-\bar{x}_{sys}\right)}{\mu_{x}}\right]\left(\frac{\bar{z}_{sys}}{\mu_{z}}\right)$* | *$b_{yx}$* | *1* |
| *$\hat{\mu}_{pi,sys}^{\left(I\right)}=\bar{y}_{sys}\left(\frac{\mu_{z}}{\lambda_{\left(opt\right)}\bar{z}_{sys}+\left(1-\lambda_{\left(opt\right)}\right)\mu_{z}}\right)$* | *$\hat{\mu}_{pi,sys}^{\left(J\right)}=\bar{y}_{sys}\left(\frac{\lambda_{\left(opt\right)}\bar{z}_{sys}+\left(1-\lambda_{\left(opt\right)}\right)\mu_{z}}{\mu_{z}}\right)$* | *0* | $\lambda_{\left(opt\right)}$ |
| *$\hat{\mu}_{pi,sys}^{\left(L\right)}=\left[\bar{y}_{sys}+\frac{\left(\mu_{x}-\bar{x}_{sys}\right)}{\mu_{x}}\right]\left(\frac{\mu_{z}}{\lambda_{\left(opt\right)}\bar{z}_{sys}+\left(1-\lambda_{\left(opt\right)}\right)\mu_{z}}\right)$* | *$\hat{\mu}_{pi,sys}^{\left(M\right)}=\left[\bar{y}_{sys}+\frac{\left(\mu_{x}-\bar{x}_{sys}\right)}{\mu_{x}}\right]\left(\frac{\lambda_{\left(opt\right)}\bar{z}_{sys}+\left(1-\lambda_{\left(opt\right)}\right)\mu_{z}}{\mu_{z}}\right)$* | *1* | $\lambda_{\left(opt\right)}$ |
| *$\hat{\mu}_{pi,sys}^{\left(N\right)}=\left[\bar{y}_{sys}+\frac{b_{yx}\left(\mu_{x}-\bar{x}_{sys}\right)}{\mu_{x}}\right]\left(\frac{\mu_{z}}{\lambda_{\left(opt\right)}\bar{z}_{sys}+\left(1-\lambda_{\left(opt\right)}\right)\mu_{z}}\right)$* | *$\hat{\mu}_{pi,sys}^{\left(O\right)}=\left[\bar{y}_{sys}+\frac{b_{yx}\left(\mu_{x}-\bar{x}_{sys}\right)}{\mu_{x}}\right]\left(\frac{\lambda_{\left(opt\right)}\bar{z}_{sys}+\left(1-\lambda_{\left(opt\right)}\right)\mu_{z}}{\mu_{z}}\right)$* | *$b_{yx}$* | $\lambda_{\left(opt\right)}$ |

**Appendix B**

**Table B1: Descriptive Statistics for Population-I**

| $\mu_{y}=27.4909$ | $\mu_{x}=3.7272$ | $C_{y}=0.3684$ | $C_{x}=0.4094$ |
| --- | --- | --- | --- |
| $\rho_{yx}=0.4327$ | $\beta_{yx}=2.8723$ | $\beta_{2x}=2.3877$ | $H_{yx}=0.3894$ |
| $H_{x}=3.5000$ | $M_{x}=4.5000$ | $T_{x}=3.7500$ | $Q_{3x}=5.0000$ |
| $G_{x}=1.7159$ | $P_{x}=1.4746$ | $DM_{x}=3.6667$ | $D_{x}=1.5206$ |

**Table B2: Descriptive Statistics for Population-II**

| $\mu_{y}=282.6136$ | $\mu_{x}=6.9943$ | $C_{y}=0.5510$ | $C_{x}=0.4231$ |
| --- | --- | --- | --- |
| $\rho_{yx}=0.8710$ | $\beta_{yx}=45.8296$ | $\beta_{2x}=3.0923$ | $H_{yx}=1.3442$ |
| $H_{x}=6.9987$ | $M_{x}=8.0000$ | $T_{x}=7.0000$ | $Q_{3x}=9.0000$ |
| $G_{x}=3.3000$ | $P_{x}=2.9079$ | $DM_{x}=7.0555$ | $D_{x}=2.9246$ |

**Table B3: Descriptive Statistics for Population-III**

| $\mu_{y}=307.9020$ | $\mu_{x}=51.1920$ | $C_{y}=0.4618$ | $C_{x}=0.5495$ |
| --- | --- | --- | --- |
| $\rho_{yx}=0.9811$ | $\beta_{yx}=1.7680$ | $\beta_{2x}=1.6877$ | $H_{yx}=0.8246$ |
| $H_{x}=51.0000$ | $M_{x}=51.5200$ | $T_{x}=51.5000$ | $Q_{3x}=76.0000$ |
| $G_{x}=32.4932$ | $P_{x}=28.7675$ | $DM_{x}=51.1778$ | $D_{x}=28.7964$ |

**Table B4: Descriptive Statistics for Population-IV**

| $\mu_{y}=5.0543$ | $\mu_{x}=4.9599$ | $C_{y}=0.4258$ | $C_{x}=0.2716$ |
| --- | --- | --- | --- |
| $\rho_{yx}=-0.9429$ | $\beta_{yx}=-1.4931$ | $\beta_{2x}=2.8039$ | $H_{yx}=-1.5237$ |
| $H_{x}=4.9539$ | $M_{x}=4.6879$ | $T_{x}=4.9349$ | $Q_{3x}=5.8259$ |
| $G_{x}=1.5268$ | $P_{x}=1.3518$ | $DM_{x}=4.9607$ | $D_{x}=1.3531$ |

**Appendix C**

**Table C1: Special cases for modified ratio estimators**

| ***Estimators*** | ***Bias and Mean Square Error*** | ***ψ_j_’s*** |
| --- | --- | --- |
| $t_{p1,sys}^{\left(A\right)}=\bar{y}_{sys}\left(\frac{\mu_{x}}{\bar{x}_{sys}}\right)$ | $\begin{matrix} Bias\left(t_{p1,sys}^{\left(A\right)}\right)\approx\theta\mu_{y}\rho_{x}^{*}C_{x}^{2}\left(1-\sqrt{\rho_{y/x}^{*}}H_{yx}\right) \\ MSE\left(t_{p1,sys}^{\left(A\right)}\right)\approx\theta\mu_{y}^{2}\left(\rho_{y}^{*}C_{y}^{2}+\rho_{x}^{*}C_{x}^{2}\left(1-2\sqrt{\rho_{y/x}^{*}}H_{yx}\right)\right). \end{matrix}$ | $\psi_{1}=1$ |
| $t_{p2,sys}^{\left(A\right)}=\bar{y}_{sys}\left(\frac{\mu_{x}+\beta_{2x}}{\bar{x}_{sys}+\beta_{2x}}\right)$ | $\begin{matrix} Bias\left(t_{p2,sys}^{\left(A\right)}\right)\approx\theta\mu_{y}\psi_{2}\rho_{x}^{*}C_{x}^{2}\left(\psi_{2}-\sqrt{\rho_{y/x}^{*}}H_{yx}\right) \\ MSE\left(t_{p2,sys}^{\left(A\right)}\right)\approx\theta\mu_{y}^{2}\left(\rho_{y}^{*}C_{y}^{2}+\psi_{2}\rho_{x}^{*}C_{x}^{2}\left(\psi_{2}-2\sqrt{\rho_{y/x}^{*}}H_{yx}\right)\right) \end{matrix}$ | $\psi_{2}=\frac{\mu_{x}}{\mu_{x}+\beta_{2x}}$ |
| $t_{p3,sys}^{\left(A\right)}=\bar{y}_{sys}\left(\frac{\mu_{x}H_{x}+M_{x}}{\bar{x}_{sys}H_{x}+M_{x}}\right)$ | $\begin{matrix} Bias\left(t_{p3,sys}^{\left(A\right)}\right)\approx\theta\mu_{y}\psi_{3}\rho_{x}^{*}C_{x}^{2}\left(\psi_{3}-\sqrt{\rho_{y/x}^{*}}H_{yx}\right) \\ MSE\left(t_{p3,sys}^{\left(A\right)}\right)\approx\theta\mu_{y}^{2}\left(\rho_{y}^{*}C_{y}^{2}+\psi_{3}\rho_{x}^{*}C_{x}^{2}\left(\psi_{3}-2\sqrt{\rho_{y/x}^{*}}H_{yx}\right)\right) \end{matrix}$ | $\psi_{3}=\frac{\mu_{x}H_{x}}{\mu_{x}H_{x}+M_{x}}$ |
| $t_{p4,sys}^{\left(A\right)}=\bar{y}_{sys}\left(\frac{\mu_{x}G_{x}+T_{x}}{\bar{x}_{sys}G_{x}+T_{x}}\right)$ | $\begin{matrix} Bias\left(t_{p4,sys}^{\left(A\right)}\right)\approx\theta\mu_{y}\psi_{4}\rho_{x}^{*}C_{x}^{2}\left(\psi_{4}-\sqrt{\rho_{y/x}^{*}}H_{yx}\right) \\ MSE\left(t_{p4,sys}^{\left(A\right)}\right)\approx\theta\mu_{y}^{2}\left(\rho_{y}^{*}C_{y}^{2}+\psi_{4}\rho_{x}^{*}C_{x}^{2}\left(\psi_{4}-2\sqrt{\rho_{y/x}^{*}}H_{yx}\right)\right) \end{matrix}$ | *$\psi_{4}=\frac{\mu_{x}G_{x}}{\mu_{x}G_{x}+T_{x}}$* |
| $t_{p5,sys}^{\left(A\right)}=\bar{y}_{sys}\left(\frac{\mu_{x}D_{x}+\beta_{2x}}{\bar{x}_{sys}D_{x}+\beta_{2x}}\right)$ | $\begin{matrix} Bias\left(t_{p5,sys}^{\left(A\right)}\right)\approx\theta\mu_{y}\psi_{5}\rho_{x}^{*}C_{x}^{2}\left(\psi_{5}-\sqrt{\rho_{y/x}^{*}}H_{yx}\right) \\ MSE\left(t_{p5,sys}^{\left(A\right)}\right)\approx\theta\mu_{y}^{2}\left(\rho_{y}^{*}C_{y}^{2}+\psi_{5}\rho_{x}^{*}C_{x}^{2}\left(\psi_{5}-2\sqrt{\rho_{y/x}^{*}}H_{yx}\right)\right) \end{matrix}$ | $\psi_{5}=\frac{\mu_{x}D_{x}}{\mu_{x}D_{x}+\beta_{2x}}$ |
| $t_{p6,sys}^{\left(A\right)}=\bar{y}_{sys}\left(\frac{\mu_{x}D_{x}+P_{x}}{\bar{x}_{sys}D_{x}+P_{x}}\right)$ | $\begin{matrix} Bias\left(t_{p6,sys}^{\left(A\right)}\right)\approx\theta\mu_{y}\psi_{6}\rho_{x}^{*}C_{x}^{2}\left(\psi_{6}-\sqrt{\rho_{y/x}^{*}}H_{yx}\right) \\ MSE\left(t_{p6,sys}^{\left(A\right)}\right)\approx\theta\mu_{y}^{2}\left(\rho_{y}^{*}C_{y}^{2}+\psi_{6}\rho_{x}^{*}C_{x}^{2}\left(\psi_{6}-2\sqrt{\rho_{y/x}^{*}}H_{yx}\right)\right) \end{matrix}$ | $\psi_{6}=\frac{\mu_{x}D_{x}}{\mu_{x}D_{x}+P_{x}}$ |
| $t_{p7,sys}^{\left(A\right)}=\bar{y}_{sys}\left(\frac{\mu_{x}H_{x}+D_{x}}{\bar{x}_{sys}H_{x}+D_{x}}\right)$ | $\begin{matrix} Bias\left(t_{p7,sys}^{\left(A\right)}\right)\approx\theta\mu_{y}\psi_{7}\rho_{x}^{*}C_{x}^{2}\left(\psi_{7}-\sqrt{\rho_{y/x}^{*}}H_{yx}\right) \\ MSE\left(t_{p7,sys}^{\left(A\right)}\right)\approx\theta\mu_{y}^{2}\left(\rho_{y}^{*}C_{y}^{2}+\psi_{7}\rho_{x}^{*}C_{x}^{2}\left(\psi_{7}-2\sqrt{\rho_{y/x}^{*}}H_{yx}\right)\right) \end{matrix}$ | $\psi_{7}=\frac{\mu_{x}H_{x}}{\mu_{x}H_{x}+D_{x}}$ |
| $t_{p8,sys}^{\left(A\right)}=\bar{y}_{sys}\left(\frac{\mu_{x}+DM_{x}}{\bar{x}_{sys}+DM_{x}}\right)$ | $\begin{matrix} Bias\left(t_{p8,sys}^{\left(A\right)}\right)\approx\theta\mu_{y}\psi_{8}\rho_{x}^{*}C_{x}^{2}\left(\psi_{8}-\sqrt{\rho_{y/x}^{*}}H_{yx}\right) \\ MSE\left(t_{p8,sys}^{\left(A\right)}\right)\approx\theta\mu_{y}^{2}\left(\rho_{y}^{*}C_{y}^{2}+\psi_{8}\rho_{x}^{*}C_{x}^{2}\left(\psi_{8}-2\sqrt{\rho_{y/x}^{*}}H_{yx}\right)\right) \end{matrix}$ | $\psi_{8}=\frac{\mu_{x}}{\mu_{x}+DM_{x}}$ |
| $t_{p9,sys}^{\left(A\right)}=\bar{y}_{sys}\left(\frac{\mu_{x}T_{x}+Q_{3x}}{\bar{x}_{sys}T_{x}+Q_{3x}}\right)$ | $\begin{matrix} Bias\left(t_{p9,sys}^{\left(A\right)}\right)\approx\theta\mu_{y}\psi_{9}\rho_{x}^{*}C_{x}^{2}\left(\psi_{9}-\sqrt{\rho_{y/x}^{*}}H_{yx}\right) \\ MSE\left(t_{p9,sys}^{\left(A\right)}\right)\approx\theta\mu_{y}^{2}\left(\rho_{y}^{*}C_{y}^{2}+\psi_{9}\rho_{x}^{*}C_{x}^{2}\left(\psi_{9}-2\sqrt{\rho_{y/x}^{*}}H_{yx}\right)\right) \end{matrix}$ | $\psi_{9}=\frac{\mu_{x}T_{x}}{\mu_{x}T_{x}+Q_{3x}}$ |
| $t_{p10,sys}^{\left(A\right)}=\bar{y}_{sys}\left(\frac{\mu_{x}DM_{x}+T_{x}}{\bar{x}_{sys}DM_{x}+T_{x}}\right)$ | $\begin{matrix} Bias\left(t_{p10,sys}^{\left(A\right)}\right)\approx\theta\mu_{y}\psi_{10}\rho_{x}^{*}C_{x}^{2}\left(\psi_{10}-\sqrt{\rho_{y/x}^{*}}H_{yx}\right) \\ MSE\left(t_{p10,sys}^{\left(A\right)}\right)\approx\theta\mu_{y}^{2}\left(\rho_{y}^{*}C_{y}^{2}+\psi_{10}\rho_{x}^{*}C_{x}^{2}\left(\psi_{10}-2\sqrt{\rho_{y/x}^{*}}H_{yx}\right)\right) \end{matrix}$ | $\psi_{10}=\frac{\mu_{x}DM_{x}}{\mu_{x}DM_{x}+T_{x}}$ |
| $t_{p11,sys}^{\left(A\right)}=\bar{y}_{sys}\left(\frac{\mu_{x}P_{x}+Q_{3x}}{\bar{x}_{sys}P_{x}+Q_{3x}}\right)$ | $\begin{matrix} Bias\left(t_{p11,sys}^{\left(A\right)}\right)\approx\theta\mu_{y}\psi_{11}\rho_{x}^{*}C_{x}^{2}\left(\psi_{11}-\sqrt{\rho_{y/x}^{*}}H_{yx}\right) \\ MSE\left(t_{p11,sys}^{\left(A\right)}\right)\approx\theta\mu_{y}^{2}\left(\rho_{y}^{*}C_{y}^{2}+\psi_{11}\rho_{x}^{*}C_{x}^{2}\left(\psi_{11}-2\sqrt{\rho_{y/x}^{*}}H_{yx}\right)\right) \end{matrix}$ | $\psi_{11}=\frac{\mu_{x}P_{x}}{\mu_{x}P_{x}+Q_{3x}}$ |

**Table C2: Special cases for regression-cum-modified ratio estimator**

| **Estimators** | **Bias and Mean Square Error** | ***ψ_j_’s*** |
| --- | --- | --- |
| $t_{p1,sys}^{\left(F\right)}=\left(\bar{y}_{sys}+\frac{b_{yx}\left(\mu_{x}-\bar{x}_{sys}\right)}{\mu_{x}}\right)\left(\frac{\mu_{x}}{\bar{x}_{sys}}\right)$ | $\begin{matrix} Bias\left(\hat{\mu}_{p1,sys}^{\left(F\right)}\right)\approx\theta\left(\lambda v\rho_{x}^{*}C_{x}^{2}\left(\beta_{sys}-\mu_{y}\sqrt{\rho_{y/x}^{*}}H_{yx}\right)-\mu_{x}^{-1}\beta_{sys}\left(\mu_{11}^{-1}\mu_{12}-\mu_{02}^{-1}\mu_{03}\right)\right) \\ MSE\left(t_{p1,sys}^{\left(F\right)}\right)\approx\theta\left(\mu_{y}^{2}\rho_{y}^{*}C_{y}^{2}+\left(\mu_{x}b_{yx}+\mu_{y}\right)\rho_{x}^{*}C_{x}^{2}\left\{\left(\mu_{x}b_{yx}+\mu_{y}\right)-2\mu_{y}\sqrt{\rho_{y/x}^{*}}H_{yx}\right\}\right) \end{matrix}$ | $\psi_{1}=1$ |
| $t_{p2,sys}^{\left(F\right)}=\left(\bar{y}_{sys}+\frac{b_{yx}\left(\mu_{x}-\bar{x}_{sys}\right)}{\mu_{x}}\right)\left(\frac{\mu_{x}+\beta_{2x}}{\bar{x}_{sys}+\beta_{2x}}\right)$ | $\begin{matrix} Bias\left(\hat{\mu}_{p2,sys}^{\left(F\right)}\right)\approx\theta\left(\lambda v\psi_{2}\rho_{x}^{*}C_{x}^{2}\left(\beta_{sys}-\mu_{y}\sqrt{\rho_{y/x}^{*}}H_{yx}\right)-\mu_{x}^{-1}\beta_{sys}\left(\mu_{11}^{-1}\mu_{12}-\mu_{02}^{-1}\mu_{03}\right)\right) \\ MSE\left(t_{p2,sys}^{\left(F\right)}\right)\approx\theta\left(\mu_{y}^{2}\rho_{y}^{*}C_{y}^{2}+\left(\mu_{x}b_{yx}+\psi_{2}\mu_{y}\right)\rho_{x}^{*}C_{x}^{2}\left\{\left(\mu_{x}b_{yx}+\psi_{2}\mu_{y}\right)-2\mu_{y}\sqrt{\rho_{y/x}^{*}}H_{yx}\right\}\right) \end{matrix}$ | $\psi_{2}=\frac{\mu_{x}}{\mu_{x}+\beta_{2x}}$ |
| $t_{p3,sys}^{\left(F\right)}=\left(\bar{y}_{sys}+\frac{b_{yx}\left(\mu_{x}-\bar{x}_{sys}\right)}{\mu_{x}}\right)\left(\frac{\mu_{x}H_{x}+M_{x}}{\bar{x}_{sys}H_{x}+M_{x}}\right)$ | $\begin{matrix} Bias\left(\hat{\mu}_{p3,sys}^{\left(F\right)}\right)\approx\theta\left(\lambda v\psi_{3}\rho_{x}^{*}C_{x}^{2}\left(\beta_{sys}-\mu_{y}\sqrt{\rho_{y/x}^{*}}H_{yx}\right)-\mu_{x}^{-1}\beta_{sys}\left(\mu_{11}^{-1}\mu_{12}-\mu_{02}^{-1}\mu_{03}\right)\right) \\ MSE\left(t_{p3,sys}^{\left(F\right)}\right)\approx\theta\left(\mu_{y}^{2}\rho_{y}^{*}C_{y}^{2}+\left(\mu_{x}b_{yx}+\psi_{3}\mu_{y}\right)\rho_{x}^{*}C_{x}^{2}\left\{\left(\mu_{x}b_{yx}+\psi_{3}\mu_{y}\right)-2\mu_{y}\sqrt{\rho_{y/x}^{*}}H_{yx}\right\}\right) \end{matrix}$ | $\psi_{3}=\frac{\mu_{x}H_{x}}{\mu_{x}H_{x}+M_{x}}$ |
| $t_{p4,sys}^{\left(F\right)}=\left(\bar{y}_{sys}+\frac{b_{yx}\left(\mu_{x}-\bar{x}_{sys}\right)}{\mu_{x}}\right)\left(\frac{\mu_{x}G_{x}+T_{x}}{\bar{x}_{sys}G_{x}+T_{x}}\right)$ | $\begin{matrix} Bias\left(\hat{\mu}_{p4,sys}^{\left(F\right)}\right)\approx\theta\left(\lambda v\psi_{4}\rho_{x}^{*}C_{x}^{2}\left(\beta_{sys}-\mu_{y}\sqrt{\rho_{y/x}^{*}}H_{yx}\right)-\mu_{x}^{-1}\beta_{sys}\left(\mu_{11}^{-1}\mu_{12}-\mu_{02}^{-1}\mu_{03}\right)\right) \\ MSE\left(t_{p4,sys}^{\left(F\right)}\right)\approx\theta\left(\mu_{y}^{2}\rho_{y}^{*}C_{y}^{2}+\left(\mu_{x}b_{yx}+\psi_{4}\mu_{y}\right)\rho_{x}^{*}C_{x}^{2}\left\{\left(\mu_{x}b_{yx}+\psi_{4}\mu_{y}\right)-2\mu_{y}\sqrt{\rho_{y/x}^{*}}H_{yx}\right\}\right) \end{matrix}$ | $\psi_{4}=\frac{\mu_{x}G_{x}}{\mu_{x}G_{x}+T_{x}}$ |
| $t_{p5,sys}^{\left(F\right)}=\left(\bar{y}_{sys}+\frac{b_{yx}\left(\mu_{x}-\bar{x}_{sys}\right)}{\mu_{x}}\right)\left(\frac{\mu_{x}D_{x}+\beta_{2x}}{\bar{x}_{sys}D_{x}+\beta_{2x}}\right)$ | $\begin{matrix} Bias\left(\hat{\mu}_{p5,sys}^{\left(F\right)}\right)\approx\theta\left(\lambda v\psi_{5}\rho_{x}^{*}C_{x}^{2}\left(\beta_{sys}-\mu_{y}\sqrt{\rho_{y/x}^{*}}H_{yx}\right)-\mu_{x}^{-1}\beta_{sys}\left(\mu_{11}^{-1}\mu_{12}-\mu_{02}^{-1}\mu_{03}\right)\right) \\ MSE\left(t_{p5,sys}^{\left(F\right)}\right)\approx\theta\left(\mu_{y}^{2}\rho_{y}^{*}C_{y}^{2}+\left(\mu_{x}b_{yx}+\psi_{5}\mu_{y}\right)\rho_{x}^{*}C_{x}^{2}\left\{\left(\mu_{x}b_{yx}+\psi_{5}\mu_{y}\right)-2\mu_{y}\sqrt{\rho_{y/x}^{*}}H_{yx}\right\}\right) \end{matrix}$ | $\psi_{5}=\frac{\mu_{x}D_{x}}{\mu_{x}D_{x}+\beta_{2x}}$ |
| $t_{p6,sys}^{\left(F\right)}=\left(\bar{y}_{sys}+\frac{b_{yx}\left(\mu_{x}-\bar{x}_{sys}\right)}{\mu_{x}}\right)\left(\frac{\mu_{x}D_{x}+P_{x}}{\bar{x}_{sys}D_{x}+P_{x}}\right)$ | $\begin{matrix} Bias\left(\hat{\mu}_{p6,sys}^{\left(F\right)}\right)\approx\theta\left(\lambda v\psi_{6}\rho_{x}^{*}C_{x}^{2}\left(\beta_{sys}-\mu_{y}\sqrt{\rho_{y/x}^{*}}H_{yx}\right)-\mu_{x}^{-1}\beta_{sys}\left(\mu_{11}^{-1}\mu_{12}-\mu_{02}^{-1}\mu_{03}\right)\right) \\ MSE\left(t_{p6,sys}^{\left(F\right)}\right)\approx\theta\left(\mu_{y}^{2}\rho_{y}^{*}C_{y}^{2}+\left(\mu_{x}b_{yx}+\psi_{6}\mu_{y}\right)\rho_{x}^{*}C_{x}^{2}\left\{\left(\mu_{x}b_{yx}+\psi_{6}\mu_{y}\right)-2\mu_{y}\sqrt{\rho_{y/x}^{*}}H_{yx}\right\}\right) \end{matrix}$ | $\psi_{6}=\frac{\mu_{x}D_{x}}{\mu_{x}D_{x}+P_{x}}$ |
| $t_{p7,sys}^{\left(F\right)}=\left(\bar{y}_{sys}+\frac{b_{yx}\left(\mu_{x}-\bar{x}_{sys}\right)}{\mu_{x}}\right)\left(\frac{\mu_{x}H_{x}+D_{x}}{\bar{x}_{sys}H_{x}+D_{x}}\right)$ | $\begin{matrix} Bias\left(\hat{\mu}_{p7,sys}^{\left(F\right)}\right)\approx\theta\left(\lambda v\psi_{7}\rho_{x}^{*}C_{x}^{2}\left(\beta_{sys}-\mu_{y}\sqrt{\rho_{y/x}^{*}}H_{yx}\right)-\mu_{x}^{-1}\beta_{sys}\left(\mu_{11}^{-1}\mu_{12}-\mu_{02}^{-1}\mu_{03}\right)\right) \\ MSE\left(t_{p7,sys}^{\left(F\right)}\right)\approx\theta\left(\mu_{y}^{2}\rho_{y}^{*}C_{y}^{2}+\left(\mu_{x}b_{yx}+\psi_{7}\mu_{y}\right)\rho_{x}^{*}C_{x}^{2}\left\{\left(\mu_{x}b_{yx}+\psi_{7}\mu_{y}\right)-2\mu_{y}\sqrt{\rho_{y/x}^{*}}H_{yx}\right\}\right) \end{matrix}$ | $\psi_{7}=\frac{\mu_{x}H_{x}}{\mu_{x}H_{x}+D_{x}}$ |
| $t_{p8,sys}^{\left(F\right)}=\left(\bar{y}_{sys}+\frac{b_{yx}\left(\mu_{x}-\bar{x}_{sys}\right)}{\mu_{x}}\right)\left(\frac{\mu_{x}+DM_{x}}{\bar{x}_{sys}+DM_{x}}\right)$ | $\begin{matrix} Bias\left(\hat{\mu}_{p8,sys}^{\left(F\right)}\right)\approx\theta\left(\lambda v\psi_{8}\rho_{x}^{*}C_{x}^{2}\left(\beta_{sys}-\mu_{y}\sqrt{\rho_{y/x}^{*}}H_{yx}\right)-\mu_{x}^{-1}\beta_{sys}\left(\mu_{11}^{-1}\mu_{12}-\mu_{02}^{-1}\mu_{03}\right)\right) \\ MSE\left(t_{p8,sys}^{\left(F\right)}\right)\approx\theta\left(\mu_{y}^{2}\rho_{y}^{*}C_{y}^{2}+\left(\mu_{x}b_{yx}+\psi_{8}\mu_{y}\right)\rho_{x}^{*}C_{x}^{2}\left\{\left(\mu_{x}b_{yx}+\psi_{8}\mu_{y}\right)-2\mu_{y}\sqrt{\rho_{y/x}^{*}}H_{yx}\right\}\right) \end{matrix}$ | $\psi_{8}=\frac{\mu_{x}}{\mu_{x}+DM_{x}}$ |
| $t_{p9,sys}^{\left(F\right)}=\left(\bar{y}_{sys}+\frac{b_{yx}\left(\mu_{x}-\bar{x}_{sys}\right)}{\mu_{x}}\right)\left(\frac{\mu_{x}T_{x}+Q_{3}}{\bar{x}_{sys}T_{x}+Q_{3}}\right)$ | $\begin{matrix} Bias\left(\hat{\mu}_{p9,sys}^{\left(F\right)}\right)\approx\theta\left(\lambda v\psi_{9}\rho_{x}^{*}C_{x}^{2}\left(\beta_{sys}-\mu_{y}\sqrt{\rho_{y/x}^{*}}H_{yx}\right)-\mu_{x}^{-1}\beta_{sys}\left(\mu_{11}^{-1}\mu_{12}-\mu_{02}^{-1}\mu_{03}\right)\right) \\ MSE\left(t_{p9,sys}^{\left(F\right)}\right)\approx\theta\left(\mu_{y}^{2}\rho_{y}^{*}C_{y}^{2}+\left(\mu_{x}b_{yx}+\psi_{9}\mu_{y}\right)\rho_{x}^{*}C_{x}^{2}\left\{\left(\mu_{x}b_{yx}+\psi_{9}\mu_{y}\right)-2\mu_{y}\sqrt{\rho_{y/x}^{*}}H_{yx}\right\}\right) \end{matrix}$ | $\psi_{9}=\frac{\mu_{x}T_{x}}{\mu_{x}T_{x}+Q_{3x}}$ |
| $t_{p10,sys}^{\left(F\right)}=\left(\bar{y}_{sys}+\frac{b_{yx}\left(\mu_{x}-\bar{x}_{sys}\right)}{\mu_{x}}\right)\left(\frac{\mu_{x}DM_{x}+T_{x}}{\bar{x}_{sys}DM_{x}+T_{x}}\right)$ | $\begin{matrix} Bias\left(\hat{\mu}_{p10,sys}^{\left(F\right)}\right)\approx\theta\left(\lambda v\psi_{10}\rho_{x}^{*}C_{x}^{2}\left(\beta_{sys}-\mu_{y}\sqrt{\rho_{y/x}^{*}}H_{yx}\right)-\mu_{x}^{-1}\beta_{sys}\left(\mu_{11}^{-1}\mu_{12}-\mu_{02}^{-1}\mu_{03}\right)\right) \\ MSE\left(t_{p10,sys}^{\left(F\right)}\right)\approx\theta\left(\mu_{y}^{2}\rho_{y}^{*}C_{y}^{2}+\left(\mu_{x}b_{yx}+\psi_{10}\mu_{y}\right)\rho_{x}^{*}C_{x}^{2}\left\{\left(\mu_{x}b_{yx}+\psi_{10}\mu_{y}\right)-2\mu_{y}\sqrt{\rho_{y/x}^{*}}H_{yx}\right\}\right) \end{matrix}$ | $\psi_{10}=\frac{\mu_{x}DM_{x}}{\mu_{x}DM_{x}+T_{x}}$ |
| $t_{p11,sys}^{\left(F\right)}=\left(\bar{y}_{sys}+\frac{b_{yx}\left(\mu_{x}-\bar{x}_{sys}\right)}{\mu_{x}}\right)\left(\frac{\mu_{x}P_{x}+Q_{3x}}{\bar{x}_{sys}P_{x}+Q_{3x}}\right)$ | $\begin{matrix} Bias\left(\hat{\mu}_{p11,sys}^{\left(F\right)}\right)\approx\theta\left(\lambda v\psi_{11}\rho_{x}^{*}C_{x}^{2}\left(\beta_{sys}-\mu_{y}\sqrt{\rho_{y/x}^{*}}H_{yx}\right)-\mu_{x}^{-1}\beta_{sys}\left(\mu_{11}^{-1}\mu_{12}-\mu_{02}^{-1}\mu_{03}\right)\right) \\ MSE\left(t_{p11,sys}^{\left(F\right)}\right)\approx\theta\left(\mu_{y}^{2}\rho_{y}^{*}C_{y}^{2}+\left(\mu_{x}b_{yx}+\psi_{11}\mu_{y}\right)\rho_{x}^{*}C_{x}^{2}\left\{\left(\mu_{x}b_{yx}+\psi_{11}\mu_{y}\right)-2\mu_{y}\sqrt{\rho_{y/x}^{*}}H_{yx}\right\}\right) \end{matrix}$ | $\psi_{11}=\frac{\mu_{x}P_{x}}{\mu_{x}P_{x}+Q_{3x}}$ |

**Appendix D**

**Table D1: Absolute Biases of (**$\hat{\mu}_{pi,sys}^{\left(A\right)}$**) Estimators**

| **Special Cases** | **Population-I** | **Population-II** | **Population-III** | **Population-IV** |
| --- | --- | --- | --- | --- |
| $\hat{\mu}_{p1,sys}^{\left(A\right)}$ | 0.1774 | 0.0192 | 0.0047 | 0.0702 |
| $\hat{\mu}_{p2,sys}^{\left(A\right)}$ | 0.1144 | 0.0297 | 0.0031 | 0.0173 |
| $\hat{\mu}_{p3,sys}^{\left(A\right)}$ | 0.1123 | 0.0291 | 0.0029 | 0.0170 |
| $\hat{\mu}_{p4,sys}^{\left(A\right)}$ | 0.1167 | 0.0301 | 0.0033 | 0.0178 |
| $\hat{\mu}_{p5,sys}^{\left(A\right)}$ | 0.1120 | 0.0293 | 0.0029 | 0.0171 |
| $\hat{\mu}_{p6,sys}^{\left(A\right)}$ | 0.1191 | 0.0307 | 0.0037 | 0.0189 |
| $\hat{\mu}_{p7,sys}^{\left(A\right)}$ | 0.1197 | 0.0309 | 0.0038 | 0.0189 |
| $\hat{\mu}_{p8,sys}^{\left(A\right)}$ | 0.1094 | 0.0287 | 0.0021 | 0.0151 |
| $\hat{\mu}_{p9,sys}^{\left(A\right)}$ | 0.1451 | 0.0301 | 0.0023 | 0.0167 |
| $\hat{\mu}_{p10,sys}^{\left(A\right)}$ | 0.1091 | 0.0284 | 0.0020 | 0.0150 |
| $\hat{\mu}_{p11,sys}^{\left(A\right)}$ | 0.1107 | 0.0289 | 0.0026 | 0.0169 |

**Table D2: Percentage Relative Efficiencies of (**$\hat{\mu}_{pi,sys}^{\left(A\right)}$**) Estimators**

| **Special Cases** | **Population-I** | **Population-II** | **Population-III** | **Population-IV** |
| --- | --- | --- | --- | --- |
| $\hat{\mu}_{p1,sys}^{\left(A\right)}$ | 103.5622 | 309.6681 | 1938.1387 | 39.19116 |
| $\hat{\mu}_{p2,sys}^{\left(A\right)}$ | 402.5696 | 281.0822 | 1553.2720 | 504.3255 |
| $\hat{\mu}_{p3,sys}^{\left(A\right)}$ | 520.4845 | 319.9596 | 1745.0490 | 522.5634 |
| $\hat{\mu}_{p4,sys}^{\left(A\right)}$ | 343.5898 | 268.1049 | 1461.5170 | 541.4378 |
| $\hat{\mu}_{p5,sys}^{\left(A\right)}$ | 328.2414 | 358.2312 | 1744.8230 | 517.5424 |
| $\hat{\mu}_{p6,sys}^{\left(A\right)}$ | 554.9469 | 335.0307 | 1352.7350 | 451.5342 |
| $\hat{\mu}_{p7,sys}^{\left(A\right)}$ | 357.9247 | 373.8497 | 1349.9888 | 448.6512 |
| $\hat{\mu}_{p8,sys}^{\left(A\right)}$ | 279.3721 | 183.8654 | 3357.9630 | 687.9685 |
| $\hat{\mu}_{p9,sys}^{\left(A\right)}$ | 315.0884 | 180.1802 | 2131.7850 | 531.8683 |
| $\hat{\mu}_{p10,sys}^{\left(A\right)}$ | 169.4997 | 175.6728 | 3404.0180 | 695.4356 |
| $\hat{\mu}_{p11,sys}^{\left(A\right)}$ | 454.2599 | 260.3858 | 1976.6863 | 551.1536 |

**Table D3: Absolute Biases of (**$\hat{\mu}_{pi,sys}^{\left(F\right)}$**) Estimators**

| **Special Cases** | **Population-I** | **Population-II** | **Population-III** | **Population-IV** |
| --- | --- | --- | --- | --- |
| $\hat{\mu}_{p1,sys}^{\left(F\right)}$ | 0.1771 | 0.0316 | 0.0052 | 0.0206 |
| $\hat{\mu}_{p2,sys}^{\left(F\right)}$ | 0.1612 | 0.0311 | 0.0042 | 0.0192 |
| $\hat{\mu}_{p3,sys}^{\left(F\right)}$ | 0.1601 | 0.0306 | 0.0040 | 0.0181 |
| $\hat{\mu}_{p4,sys}^{\left(F\right)}$ | 0.1662 | 0.0308 | 0.0044 | 0.0195 |
| $\hat{\mu}_{p5,sys}^{\left(F\right)}$ | 0.1607 | 0.0303 | 0.0041 | 0.0184 |
| $\hat{\mu}_{p6,sys}^{\left(F\right)}$ | 0.1615 | 0.0307 | 0.0043 | 0.0194 |
| $\hat{\mu}_{p7,sys}^{\left(F\right)}$ | 0.1662 | 0.0308 | 0.0044 | 0.0195 |
| $\hat{\mu}_{p8,sys}^{\left(F\right)}$ | 0.1586 | 0.0300 | 0.0035 | 0.0147 |
| $\hat{\mu}_{p9,sys}^{\left(F\right)}$ | 0.1589 | 0.0301 | 0.0037 | 0.0164 |
| $\hat{\mu}_{p10,sys}^{\left(F\right)}$ | 0.1583 | 0.0299 | 0.0034 | 0.0123 |
| $\hat{\mu}_{p11,sys}^{\left(F\right)}$ | 0.1537 | 0.0297 | 0.0029 | 0.0090 |

**Table D4: Percentage Relative Efficiencies of (**$\hat{\mu}_{pi,sys}^{\left(F\right)}$**) Estimators**

| **Special Cases** | **Population-I** | **Population-II** | **Population-III** | **Population-IV** |
| --- | --- | --- | --- | --- |
| $\hat{\mu}_{p1,sys}^{\left(F\right)}$ | 173.2377 | 214.7512 | 1003.5780 | 128.3857 |
| $\hat{\mu}_{p2,sys}^{\left(F\right)}$ | 168.2544 | 222.9106 | 1141.6290 | 142.2851 |
| $\hat{\mu}_{p3,sys}^{\left(F\right)}$ | 192.2305 | 270.4547 | 1270.8318 | 147.9625 |
| $\hat{\mu}_{p4,sys}^{\left(F\right)}$ | 112.4480 | 261.6317 | 1079.7730 | 139.8237 |
| $\hat{\mu}_{p5,sys}^{\left(F\right)}$ | 164.9394 | 269.6873 | 1270.6790 | 147.2947 |
| $\hat{\mu}_{p6,sys}^{\left(F\right)}$ | 138.8985 | 238.1584 | 1005.2750 | 131.2748 |
| $\hat{\mu}_{p7,sys}^{\left(F\right)}$ | 175.8107 | 217.4337 | 1004.4160 | 130.4351 |
| $\hat{\mu}_{p8,sys}^{\left(F\right)}$ | 111.3296 | 285.2871 | 2445.3300 | 172.1187 |
| $\hat{\mu}_{p9,sys}^{\left(F\right)}$ | 150.6530 | 281.5635 | 1533.2180 | 151.0010 |
| $\hat{\mu}_{p10,sys}^{\left(F\right)}$ | 187.4838 | 297.0094 | 2484.6470 | 187.7186 |
| $\hat{\mu}_{p11,sys}^{\left(F\right)}$ | 293.2391 | 362.6474 | 2761.1572 | 244.8603 |

**Appendix E: Nomenclatures**

**Table E: Robust Measures of the Auxiliary Variable**

|  | Downton’s Method |
| --- | --- |
| $DM_{x}=\left(D_{1}+D_{2}+,...,+D_{9}\right)/9$ $D_{i}=i\left(\frac{n+1}{10}\right)^{th}\mathrm{value}$ where *D_i_* is the *i^th^* decile based on the auxiliary variable | Decile Mean |
|  | Gini’s mean difference |
| $\begin{matrix} HL_{x}=median\left(X_{j}-X_{k}\right)/2 \\ 1\leq j\leq k\leq N \end{matrix}$ | Hodges-Lehmann |
| $M_{x}=\left(X_{max}-X_{min}\right)/2$ where *X_max_* and *X_min_* are the maximum and the minimum values of the auxiliary variable | Mid-range |
|  | Probability weighted moments |
| $T_{x}=\left(Q_{1x}+2Q_{2x}+Q_{3x}\right)/4$ where *Q_1x_*, *Q_3x_* and *Q_2x_* are the lower, upper and middle Quartiles of the auxiliary variable | Tri-mean of the auxiliary variable |
| $Q_{3x}=3\left(\frac{n+1}{4}\right)^{th}observation$ | Upper Quartile |
